# Supplementary figures and images for: H2B.V demarcates divergent strand-switch regions, some tDNA loci, and genome compartments in Trypanosoma cruzi and affects parasite differentiation and host cell invasion
Source: PLoS Pathog. 2022 Feb 18;18(2):e1009694. doi: 10.1371/journal.ppat.1009694 (PMC8893665; doi:10.1371/journal.ppat.1009694)

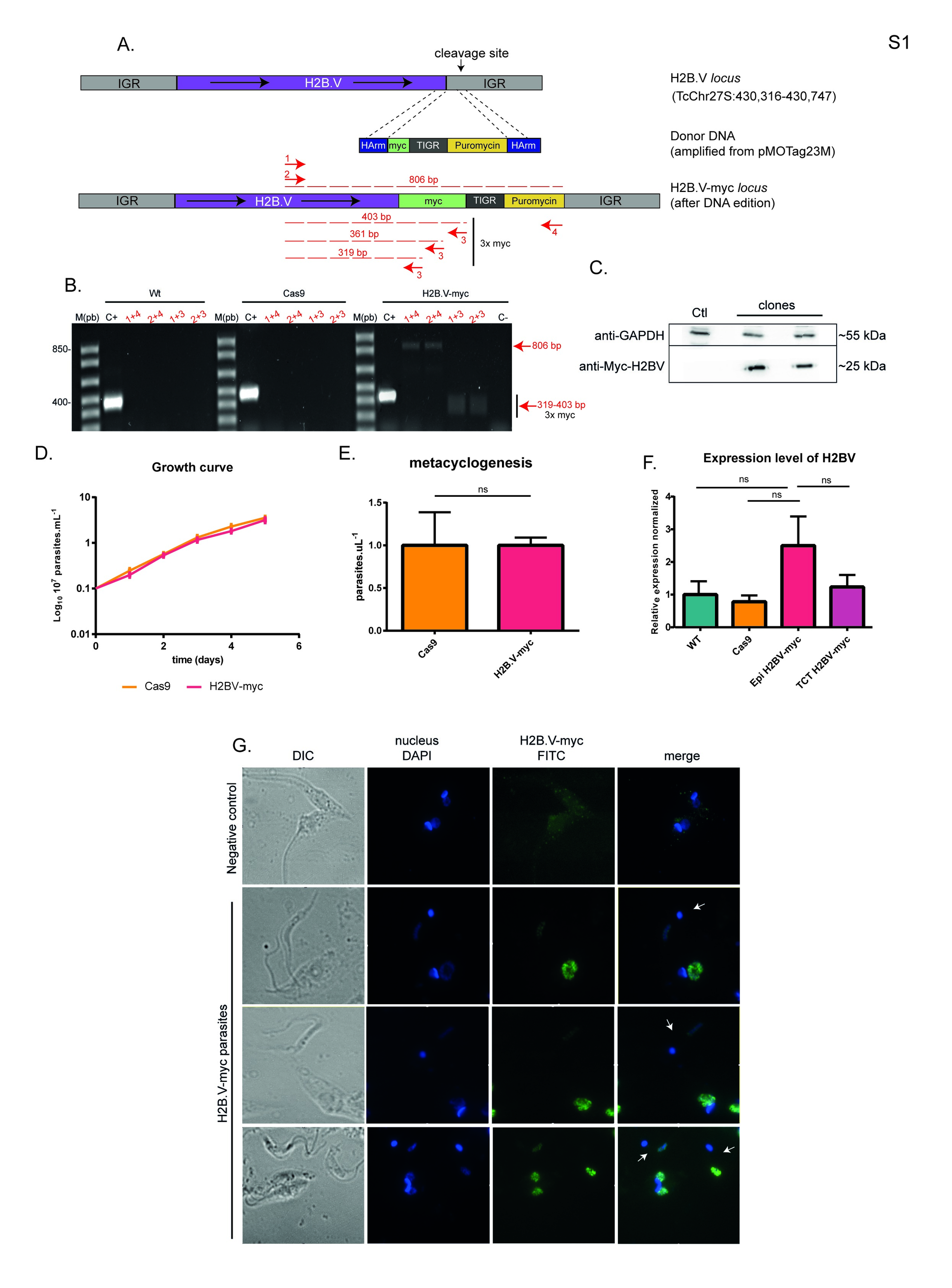

Supplement: S1 Fig — A. Schematic representation of CRISPR/Cas9 gene editing of the H2B.V gene (TcChr27-S:430,316–430,747). The small black arrow indicates the cleavage site caused by the Cas9 enzyme. IGR represents the intergenic region, and HArm represents the homologous arm. Donor DNA was amplified by PCR from the pMOTag23M plasmid using long primers. Long primer sequences are composed of 30 nucleotides corresponding to the homologous arm (5’ end of the primer) plus 20 nucleotides complementary to the plasmid (3’ end of the primer). The resulting PCR product, called donor DNA, is composed of 30 bp of the homologous arm of the H2B.V gene (in blue); 3 copies of myc sequence (in green); the T. brucei tubulin intergenic region (TIGR—dark gray); the resistance gene to puromycin (in yellow); and 30 bp of the homologous arm (in blue) from the 3’ intergenic region (just after the cleavage site). The final edited locus H2B.V–myc is illustrated at the bottom of the scheme.The red arrows indicate the primers used to evaluate genomic edition in both alleles (Esmeraldo-like and Non Esmeraldo-like haplotypes). B. Agarose 1% gel showing that insertion of myc-tagged occurred in both alleles of the CL Brener strain. An insert of ~800 bp (primers 1+4 and 2+4), and 350–400 (1+3 and 2+3) were detected. Primers 1 and 2 were designed to be allele-specific. Genomic DNA from wt and Cas9 parasites were used as a negative control for amplification of myc and puromycin gene (C-) and as a positive control (C+) for amplification of H2B gene (primers H2Bc_F and H2Bc_R). C. Western blot assays confirm the expression of H2B.V-myc in epimastigotes maintained with puromycin (10–30 μg/mL). Untransfected parasites were used as a control (Ctl). Phenotypic evaluation of H2B.V-myc parasites: D. Growth curves (in log10) for Cas9 and H2B.V-myc parasites; E. The number of metacyclic trypomastigotes from Cas9 and H2B.V-myc parasites were counted in the RPMI supernatant after 8 days. The values were normalized to their respect [file ppat.1009694.s001.tif]

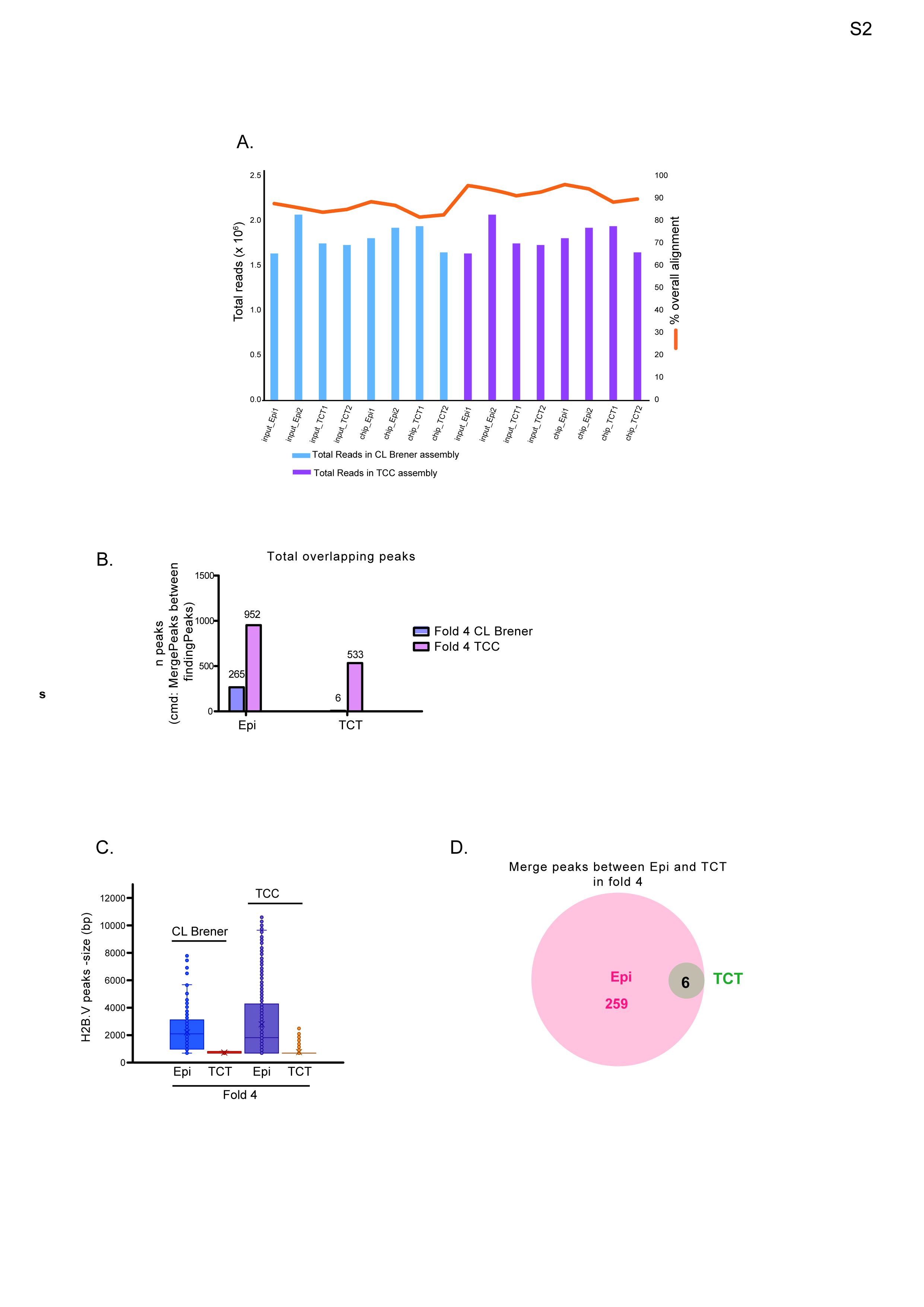

Supplement: S2 Fig — A. Total reads and overall alignment of input and H2B.V- ChIP samples in the CL Brener Esmeraldo-like haplotype (release 32) and TCC (release 44) assemblies. B. H2B.V peaks identified in epimastigotes and TCTs using a peak calling algorithm available in HOMER [27] considering a fold enrichment of 4 (default) and a required Poisson p-value over input = 1.00e-04. C. Comparison of peak (fold 4) width (in bp) among life forms and genome mapping. (D) Venn diagrams (available online at https://www.meta-chart.com/) comparing common and different peaks at fold 4 between life forms. (TIF) [file ppat.1009694.s002.tif]

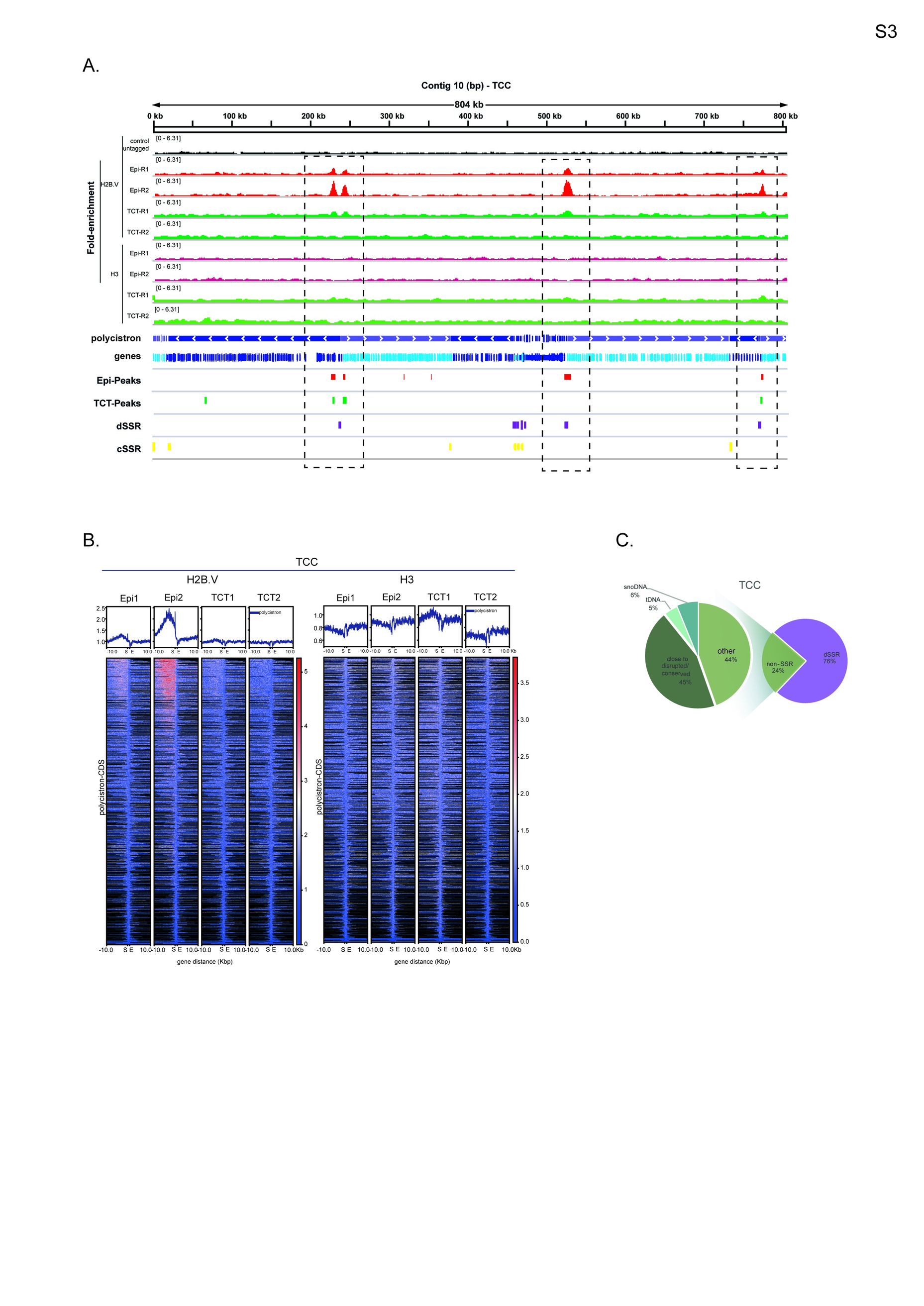

Supplement: S3 Fig — A. IGV snapshot of H2B.V and H3 enrichment at contig PRFC000010. Note that H2B.V, but not H3, is enriched at dSSR (rectangles with interrupted lines). Blue bar arrows indicate the transcription direction in each polycistron. Genes in the same polycistron are stained with the same color. Red and green bars represent, respectively, H2B.V-peaks (fold 4) obtained by HOMER in epimastigote and TCT life forms. Purple and yellow bars represent dSSRs and cSSRs, respectively. B. Heatmap plots and k-mean clustering of H2B.V and H3 ChIP-seq signals at CDS polycistrons (deeptools—scale region function) and their 10-kb upstream and downstream regions, mapped to the TCC assembly. Black regions represent polycistrons that are located at the border of the contigs and therefore have either no upstream or downstream regions. C. Distribution of H2B.V peaks (fold 4) in the 20 longer contigs (PRFC000001 to PRFC000020) of the TCC assembly. (TIF) [file ppat.1009694.s003.tif]

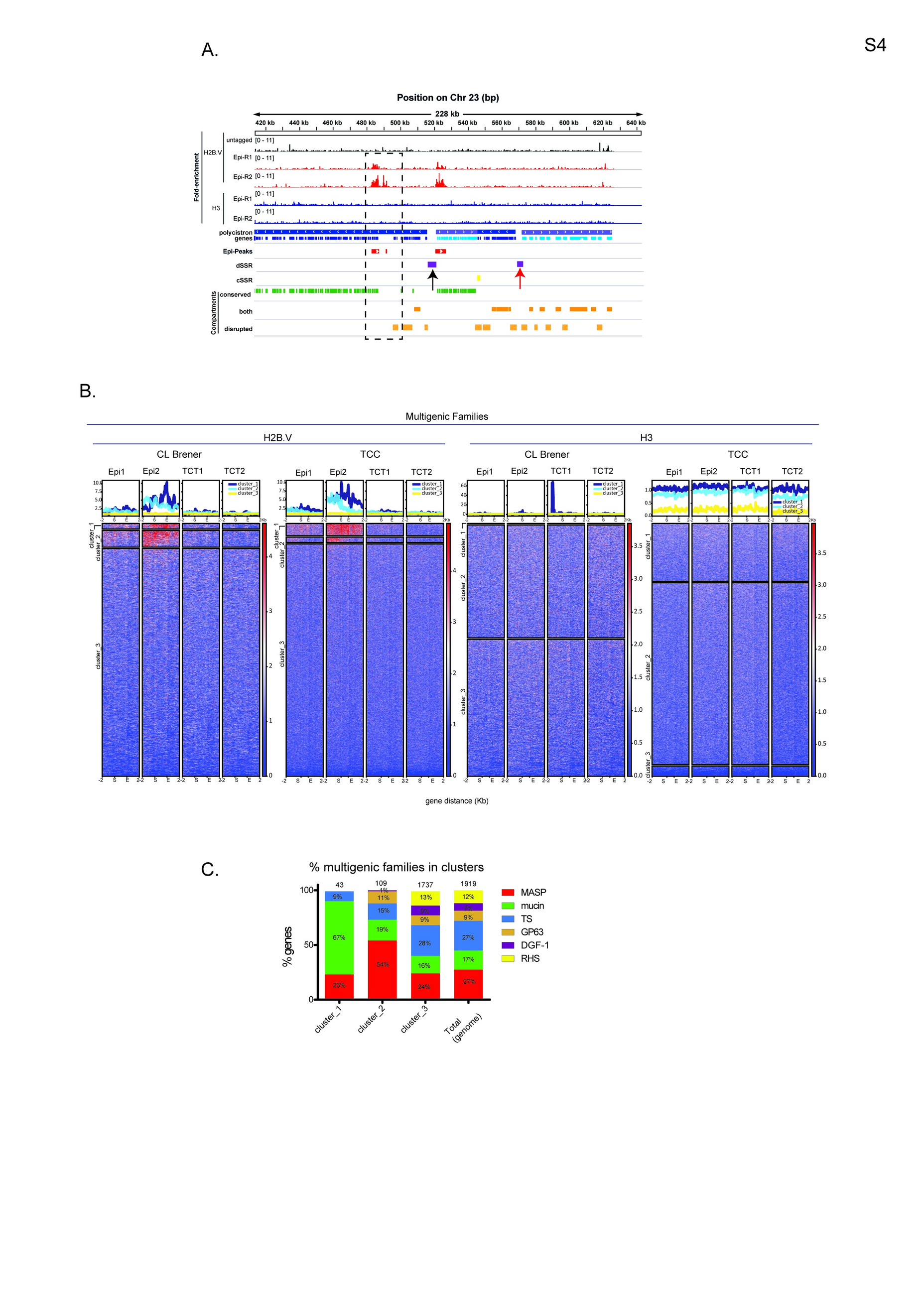

Supplement: S4 Fig — A. IGV snapshot of H2B.V and H3 reads in TcChr23-S showing an enrichment of H2B.V between the conserved (green) and disrupted (orange) genome compartments (rectangle with interrupted lines) and two distinct patterns at dSSRs (black and red arrows). Blue bar arrows indicate the transcription direction in each polycistron. Genes in the same polycistron are stained with the same color. Red and green bars represent, respectively, H2B.V -peaks (fold 4) obtained by HOMER in epimastigote and TCT life forms. Purple and yellow bars represent dSSRs and cSSRs, respectively. Black and red arrows indicate dSSRs with or without H2B.V enrichment, respectively. B. Heatmap plots and k-means clustering of H2B.V and H3 ChIP-seq signals in multigenic family members (deeptools—scale region function) and their 2-kb upstream and downstream regions mapped in the CL BrenerCL Brener- Esmeraldo-like and TCC assembly. C. Distribution of multigenic family members in clusters 1 to 3 described in B (dark blue, light blue and yellow lines in the summary plot). Note that the distribution of these genes in clusters 1 and 2 differs from the expected genome distribution (last bar). (TIF) [file ppat.1009694.s004.tif]

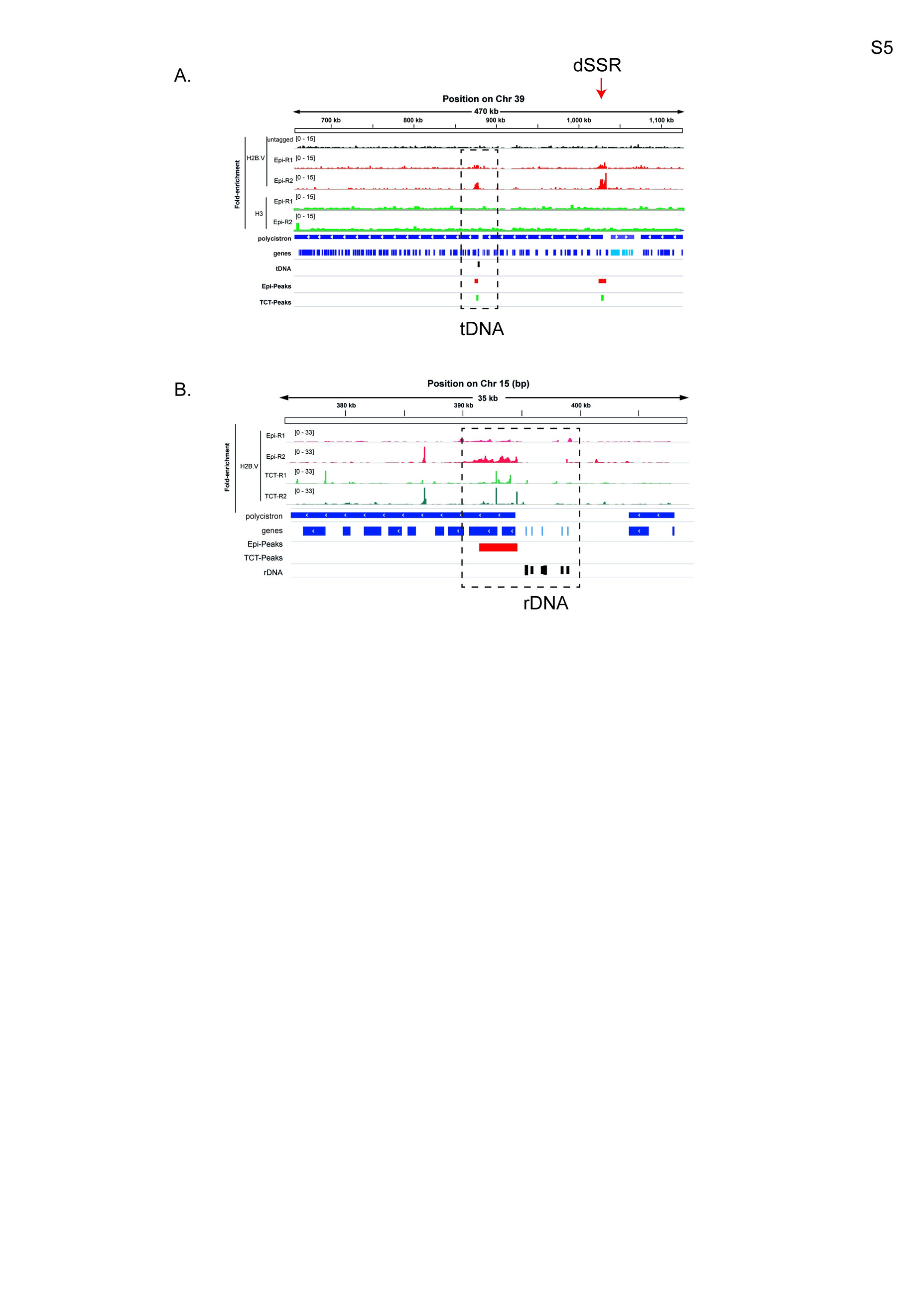

Supplement: S5 Fig — IGV snapshot of H2B.V enrichment at tDNA loci, dSSR (A) and at rDNA (B) in the CL Brener Esmeraldo-like assembly. The enrichment at the indicated feature is highlighted by a rectangle with interrupted lines. Blue bars arrows indicate the transcription direction in each polycistron. Genes in the same polycistron are stained with the same color. Red and green bars represent, respectively, H2B.V -peaks (fold 4) obtained by HOMER in epimastigote and TCT life forms. Black bars indicate tDNA or rDNA. (TIF) [file ppat.1009694.s005.tif]

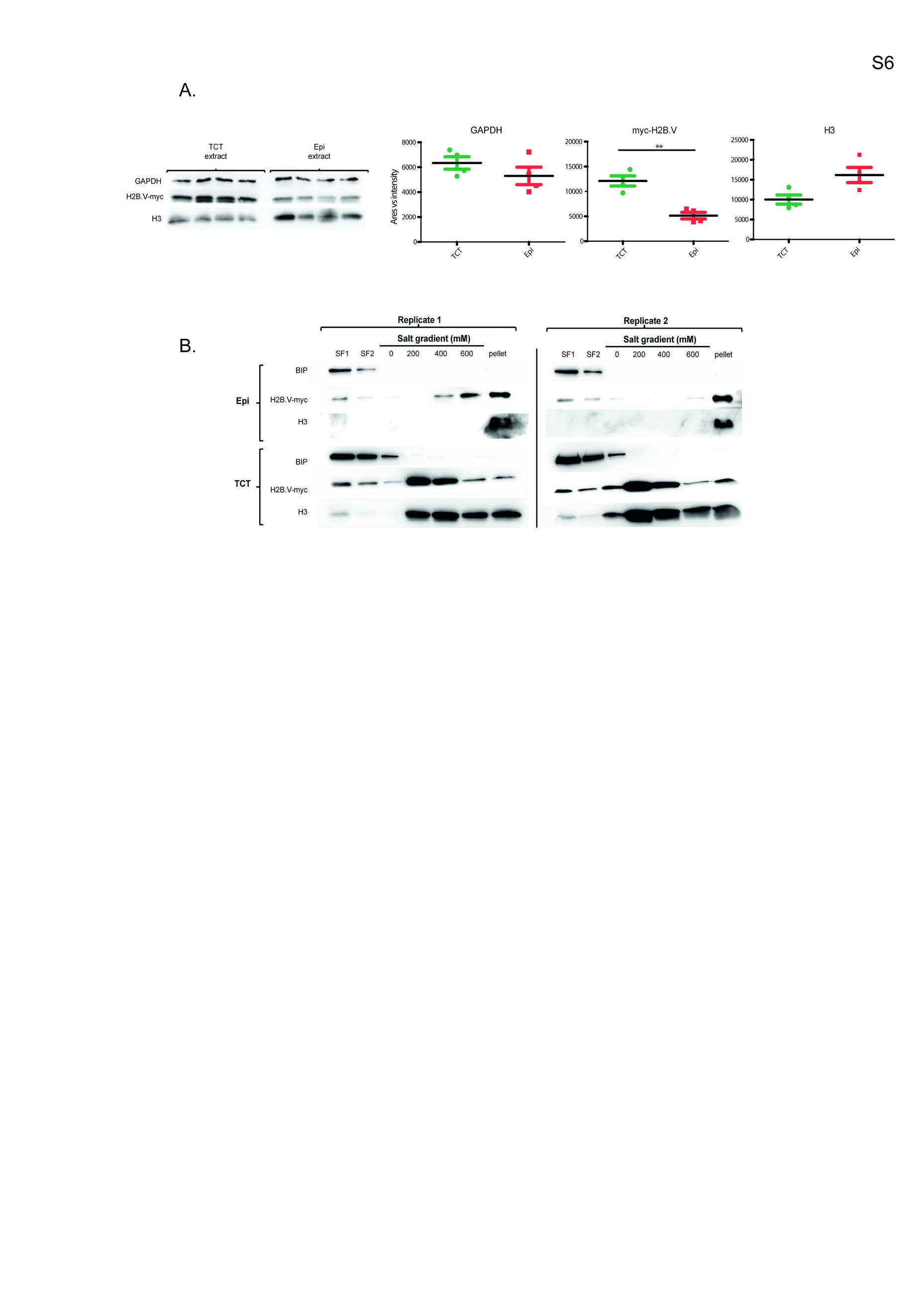

Supplement: S6 Fig — WCE of TCTs and epimastigotes (2x10^6^ parasites in quadruplicates) were probed against GAPDH (housekeeping gene), myc (H2B.V) and histone H3. Right, bands were quantified using ImageJ and plotted in Prisma. ** t-test (p-value < 0,005). B. Chromatin sequential salt extraction from epimastigotes and TCTs in biological duplicates were probed against BiP (cytosol marker), myc (H2B.V) and histone H3. SF1 –soluble fraction 1 and SF2 –soluble fraction 2 and pellet as insoluble sediment. The equivalent of 200 ng of DNA for epimastigotes and TCTs were fractionated in SDS-Page. (TIF) [file ppat.1009694.s006.tif]

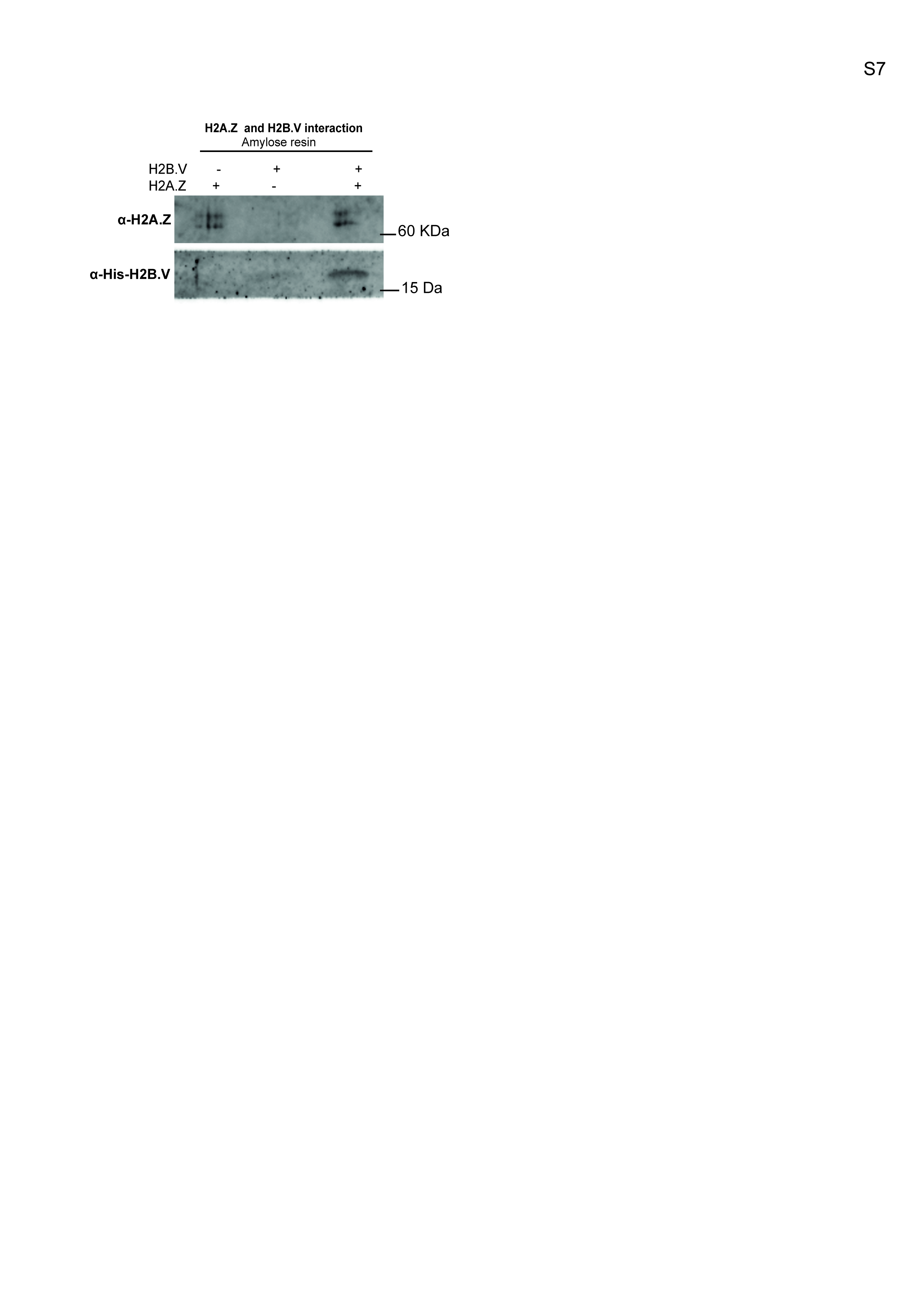

Supplement: S7 Fig — Recombinant H2A.Z-MBP and recombinant H2B.V-His were incubated together or separately with amylose resin, known to specifically interact with MBP. Eluates were fractionated in a 15% SDS-PAGE and transferred to nitrocellulose membranes. Western blot assay was performed using polyclonal antibodies anti-H2A.Z and anti-His (for H2B.V-HisTag). H2A.Z-MBP is ~60 kDa, and H2B.V-His is ~ 16.5 kDa. (TIF) [file ppat.1009694.s007.tif]

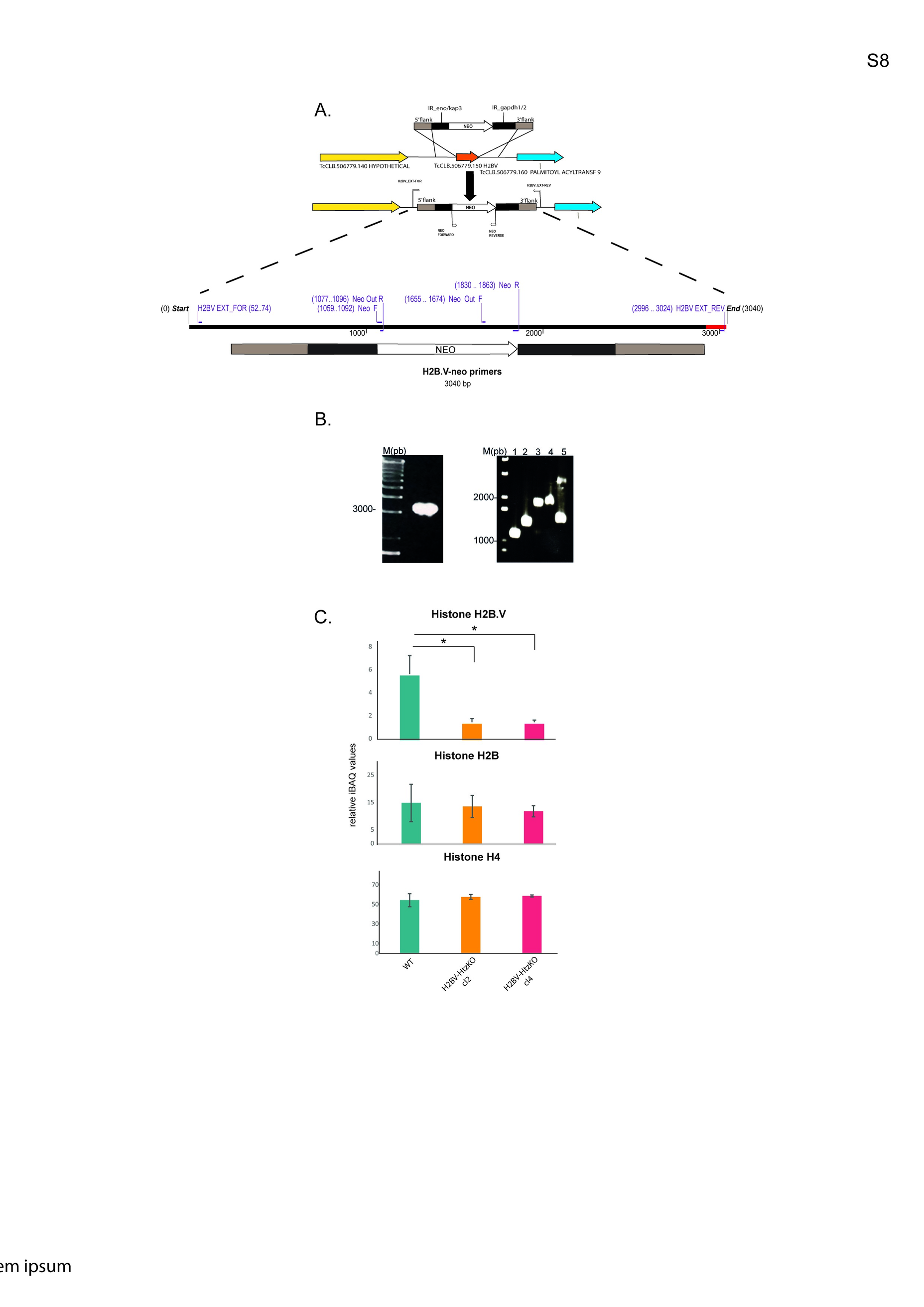

Supplement: S8 Fig — (A) Diagram of NEO cassette recombination at the H2B.V locus (Chr 27-S) in the T. cruzi CL Brener genome highlighting the location of H2B_EXT_FOR and H2B_EXT_REV primers used for amplification of the whole NEO cassette, which includes the 5’ and 3’ fragments of H2B.V. Lower, the location of primers H2B.V EXT_FOR (forward); Neo F (forward); Neo Out R (reverse); Neo Out F (forward); Neo R (reverse); and H2B.V EXT_REV (reverse) are highlighted. (B) pTc2KO-H2B.V-neo cassette insertion confirmation. Left, the 1% TAE agarose gel showing the 3045-bp amplicon from the complete NEO cassette recombined at the H2B.V locus (primers H2B_EXT_FOR and H2B_EXT_REV). Right, pTc2KO-H2B.V-neo cassette insertion confirmation in clone 4. A 1% TAE agarose gel of amplificons obtained from PCR using the following pair of primers: 1- H2B.V EXT_FOR / Neo Out R (1052 bp); 2- Neo Out F / H2B.V EXT_REV (1376 bp); 3- H2B.V EXT_FOR / Neo R (1819 bp); 4- Neo F / H2B.V EXT_REV (1966 bp); H2B.V_KpnI_forward / H2B.V_XbaI_reverse (1384 bp) for confirmation of heterozygous knockouts. M(pb)- molecular weight marker in bp (1 Kb Plus Ladder–Invitrogen). (C) Relative abundance of histones in H2B.V-HztKO and wild-type parasites. Total basic extracts from H2B.V-HtzKO and wild-type parasites were evaluated by label-free quantitative proteomics. iBAQ values were obtained from each sample (in biological triplicates), and the relative values were considered. Unpaired t-test (* p-value < 0.05). (TIF) [file ppat.1009694.s008.tif]

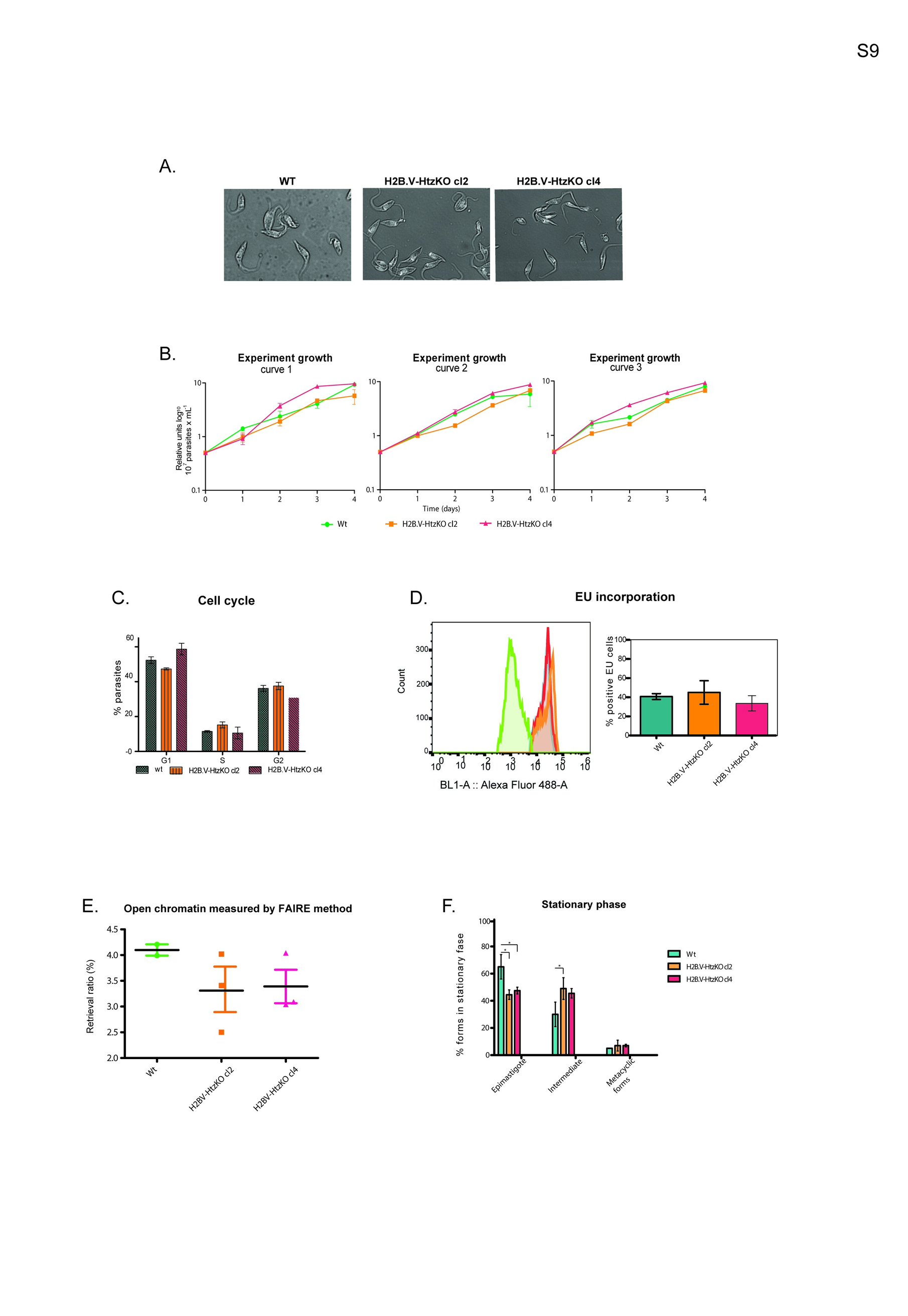

Supplement: S9 Fig — A. Phase contrast images of H2B.V-HtzKO, and wild-type parasites. B. Biological replicates of growth curves (in log10) for wild-type and H2B.V-HtzKO (cl 2 and 4) parasites. C. Percentage of cell cycle phases in H2B.V-HtzKO clones and wild-type parasites. Bars represent the average from biological triplicates. Error bars represent SEM values. D. Histograms of EU-positive cells of H2B.V-HtzKOs (cl2 and 4), wild-type (orange) and unlabeled parasites (green). The mean and standard variation of the percentage of positive cells were plotted (triplicate values). One-way ANOVA of variance–unpaired-test. E. Quantitation of the open chromatin of H2B.V-HtzKOs and wild-type parasites obtained after the FAIRE protocol. The retrieval ratio refers to the percentage of open chromatin related to the total DNA content obtained for each sample. F. Percentage of parasites (epimastigote, intermediate and metacyclic trypomastigotes) in stationary phase culture of H2B.V-HtzKOs and wild-type parasites. Parasites were classified based on the position and morphology of the nucleus and kinetoplast as proposed previously [25]. The metacyclics markers GP90 and GP82 were also used to allow discrimination of life forms. (TIF) [file ppat.1009694.s009.tif]
