# Supplementary material for: H2B.V demarcates divergent strand-switch regions, some tDNA loci, and genome compartments in Trypanosoma cruzi and affects parasite differentiation and host cell invasion
Source: PLoS Pathog. 2022 Feb 18;18(2):e1009694. doi: 10.1371/journal.ppat.1009694 (PMC8893665; doi:10.1371/journal.ppat.1009694)
Supplement: Attachment — Submitted filename: answers.docx [file ppat.1009694.s015.docx]

**Dear PlosPathogens Editor,**

We thank the editors and the referees for carefully reading our manuscript and making important questions and suggestions. We have answered all questions accordingly, and we are confident that the manuscript is now more robust and clearer. We hope that this manuscript meets the high standards of the PlosPathogens Journal.

This new version includes assays suggested by the reviewers that are depicted mainly at Figures S1B, S1D-F, and S6B. For simplicity, we removed panel C from FigS5.

Please find below the answers to all referee’s questions.

Sincerely,

Julia Cunha, PhD

**Reviewer's Responses to Questions**

**Part I - Summary**
Please use this section to discuss strengths/weaknesses of study, novelty/significance, general execution and scholarship.

Reviewer #1: The manuscript presents a novel and significant study of H2B.V histone variant in T. cruzi, assessing its genome distribution by CHIP-seq of endogenously tagged (Myc) parasites, its in vitro protein partners by affinity chromatography and its functional significance by in vitro assays of hemi-KO parasites.
The genome-wide distribution of histone variant H2B.V in T. cruzi epimastigotes uncovered a specific positioning at the dSSR of polycistron (as shown in T. brucei), the boundaries between conserved and disrupted gene compartments, and tDNA putative internal polycistronic TSS mostly. Curiously, cellular trypomastigotes are depleted of the variant in those regions and no specific enrichment could be found in this stage. Finally, H2B.V in vitro protein partners identified include BDF2 and H2A.Z (previously known to overlap in the SSR of T. brucei), and 10 H2B.V specific chromatin associated proteins.
Overall, these findings confirm the T. brucei model of H2B.V function, in which pol II transcription start sites (TSSs) are occupied by unstable nucleosomes containing H2A.Z and H2BV (as well as BDF3 and H4K10ac), likely facilitating transcription initiation.
The authors also study the effect of the single copy KO of the H2B.V variant, unable to analyze the double KO for the probable gene essentiality, not surprisingly since it H2B.V and H2A.Z were already shown to be essential for cell viability in T. brucei (2005) and L. major (2013). Interestingly, they see an alteration of the differentiation and invasion rates but not proliferation, growth, or transcriptional levels in the H2B.V-KO parasites.
Finally, the current manuscript provides novel chromatin information for T. cruzi gene families relevant for host infection, showing that gene in the MUCINs and MASP (located in the disrupted compartments are more enriched in H2B.V compared to the multigenic families TS, GP63, RHS, and DGF-1, perhaps for they preferential location at the first CDS of their cognate polycistron (as discussed by the authors).

The experimental strategy, experimental design, presentation and analysis of the data are correct, and the results are sound, and reasonable for the aim of this kind of high throughput studies. Future experiments would be needed to validate the findings and investigate the influence of H2B.V in gene transcription and its molecular mechanism of action. Additionally, the text is clear, the results are correctly presented, and the discussion is fair and honestly states the limitations of the study.

Reviewer #2: This is a comprehensive contribution to the understanding of chromatin status and dynamics in Trypanosoma cruzi that may represent an important step on gene expression regulation across parasite differentiation. The authors show that the distribution of the H2B-V demarcates strategic regions of the genome of T. cruzi, which affects replicative to nonreplicative differentiation and host cell invasion. Data presented is robust and thoroughly discussed. It may be considered a major contribution to the field.

**Part II – Major Issues: Key Experiments Required for Acceptance**
Please use this section to detail the key new experiments or modifications of existing experiments that should be absolutely required to validate study conclusions.

Generally, there should be no more than 3 such required experiments or major modifications for a "Major Revision" recommendation. If more than 3 experiments are necessary to validate the study conclusions, then you are encouraged to recommend "Reject".

Reviewer #1:

1. A major concern is the emphasis made by the authors narrative on the replicative vs non-replicative states. Since replication is not the major difference between epimastigotes and trypomastigotes forms, and the functional results of this study point to stage specific roles of H2B.V and not proliferative ones, it is unclear why the authors highlight the replicative comparison. The authors need to clarify the relevance of the H2B.V replicative connection and incorporate amastigotes and metacyclic trypomastigote in the study. If that stress is just a narrative imprecision, the authors should rephrase the associated paragraphs.

**Answer.** We agree with the referees that it is a narrative imprecision. For most cases, the comparison was made between epimastigotes and TCTs, thus we could not affirm that the findings were due to the replicative capacity. Then, we have changed the associated paragraphs accordingly. When appropriated, the terms “replicative” and “non-replicative” was replaced by the epimastigote and TCTs, respectively.

2. The second major concern is about the expression of H2B.V in trypomastigotes. In De Jesus et al 2016, the authors found that H2B.V variant is notoriously overexpressed in trypomastigotes compared to epimastigotes, as mentioned in the manuscript. In the current study they show the same trend trough the quantification of myc-H2B.V expression by western blot in whole extracts. Yet, SF1C shows myc-H2B.V trypomastigote parasites (probably indicated by the arrows -although there is no reference to them in the legend or the main text-), lacking or having faint myc signal. Unfortunately, Figure SF1C is not commented in the text. Although the authors extensively discuss the conundrum of the high expression of H2B.V but no enrichment in chromatin regions (364-376), the SF1C pictures (if correct) are impressive. The authors must clarify how is the expression myc-H2B.V in the edited trypomastigote parasites using IHQ.

**Answer.** First, we must highlight that the previous enrichment of H2B.V in TCTs chromatin (found at Jesus et al 2016) was obtained using a protocol based on protein releasement upon DNAse treatment. This protocol collects the supernatant of the DNAse-treated nuclear samples, obtaining proteins with different DNA-binding affinities.

To solve the conundrum, we first hypothesize that the faint H2B.V staining in TCTs was due to the enrichment of H2B.V in a compacted chromatin region affecting antibody recognition. Thus, we performed IFA using denaturing protocols with HCl to expose different chromatin regions and evaluated if H2B.V staining would be more evident. No difference was found, suggesting that H2B.V in TCTs was not localized in an inaccessible chromatin region (data not shown).

Thus, we hypothesize that H2B.V may interact weakly with TCT’s chromatin and easily release upon experimental procedures, such as IFA and ChIP. To address this, we performed a sequential salt extraction assay, which evaluated the relative binding affinities of chromatin-associated proteins. The results indicated that H2B.V (and H3), in TCT forms, is released from chromatin in lower salt concentrations. In contrast, both histones are released with higher salt concentrations in epimastigotes, indicating that, indeed, H2B.V is weaklier bonded to TCTs chromatin. In addition, figure SF1C is cited, and arrows are explained in the legend.

Then, we changed the text as follow:

lines 237-246

*“This finding agrees with lower H2B.V staining in myc-tagged TCTs nuclei detected by IFA (S1G Fig) but contrasts with previous findings [31].*

*Thus, we hypothesize that H2B.V may weakly interact with the TCT’s chromatin and likelybe released upon experimental procedures like IFA and ChIP. To address this, we performed a chromatin sequential salt extraction assay, which evaluates the relative binding affinities of chromatin-associated proteins [37]. The results indicated that H2B.V (and H3) is released from chromatin in lower salt concentrations in TCTs than epimastigote forms, suggesting that H2B.V has a lower binding affinity to TCTs chromatin (S6B Fig).*

*As a whole, these findings…”*

3. In addition, an assessment of the expression of myc-H2B.V in the transfected cell line compared to the wild type by qRT-PCR is necessary. Also, it is unclear if the edited parasite line is homozygote for the tag, and that can be also discriminated by qRT-PCR. Although the authors explain the lack of antibodies (138 lines), allele specific PCR is possible with this type of edition, and qRT-PCR just to assess the bi-allelic expression can be done as for the KO transfectants.

**Answer.** We thank the reviewer for asking these questions. We evaluated, by qPCR, the expression levels of myc-H2B.V in our H2B.V-tagged parasites and compared the expression in wild-type cells and cells expressing the Cas9 gene. Statistical analysis indicated no significant difference between groups (Figure S1F).

In addition, we have drawn allele-specific primers to evaluate whether the parasite is homo or heterozygote for the tag. The results are depicted in Figure S1B. The myc-tag was inserted in both alleles: primers 1 and 2 discriminate the S or P alleles from Esmeraldo and Non-Esmeraldo haplotype, respectively. Amplicons from haplotypes were obtained for primers against myc and puromycin gene (presented at inserted cassette). Taken together, we conclude that the tag was inserted in both alleles. Moreover, as suggested by the second reviewer, we have evaluated if H2B.V transfected parasites behave similarly to their wild type in terms of proliferation and metacyclogenesis. The results are shown in Figures S1D and E. These results are now explained in the text:

Lines 145-151.

*“Allele-specific PCRs indicate that both alleles were edited (S1B Fig­­). The genome edition neither affects parasite growth nor the metacyclogenesis processes (S1D-E Fig­­). In addition,* *H2B.V transcript abundance in epimastigotes and TCTs do not differ from wild-type cells or parasites expressing Cas9 (S1F Fig­­). Together, these data indicated that the genomic edition at H2B.V locus confers no significant phenotypic changes.”*

Reviewer #2: (No Response)

**Part III – Minor Issues: Editorial and Data Presentation Modifications**
Please use this section for editorial suggestions as well as relatively minor modifications of existing data that would enhance clarity.

Reviewer #1:

1. I suggest changing the title entirely. The adjective “strategic” has an overly broad meaning and does not effectively communicate the findings of the study. In addition, the emphasis in the interaction with the bromodomain protein points to a finding that is not central in the manuscript and may need further proof to become meaningful in vivo and for the 2 stages analyzed.

**Answer.** Both reviewers suggested to change the title. Considering their comments, the title was changed to:

*“****H2B.V demarcates divergent strand-switch regions, some tDNA loci and genome compartments in T. cruzi and affects parasite differentiation and host cell invasion****”*

2. Line 41- …was preferentially located at divergent “TRANSCRIPCIONAL STRAND” switch regions. Insert the words in upper case or similar to be precise.

**Answer.** Done.

3. Although it is common to omit this aspect, I think is always useful to justify the selection of parasite strains for the experiments. Please comment the reason of the choice of the 2 different strains used.

**Answer.** The Y strain has been used in our lab (and from our close collaborators) in the past 10 years allowing results comparison. In fact, the identification of H2B.V differential enrichment in life forms chromatin was obtained in the Y strain. In this context, we generated single knockout parasites and pulldown assays. By the time we aimed to generate parasites expressing H2B.V-myc tagged, the Crispr-Cas9 technology was standardized for the CL Brener strain. In fact, we had in “our hands” this strain expressing the Cas9 protein. In 2017 (when this project started), T. cruzi Y strain had no sequencing information about their genome hampering ChIP-seq analysis. Thus, we opted to obtain the tagged parasite and, consequently, the Chip-seq results using the CL Brener strain. As the manuscript story is not chronologically organized, it may seem odd for the readers why we started with the CL Brener strain (Chip-seq results) and continued the experiments with the Y strain (pull-down and single knockout parasites). To clarify this fact, we added a short sentence in the manuscript explaining why we changed the CL Brener strain for Y strain, as follow:

Lines 255-256:

*“From here, all experiments were performed with the T. cruzi Y strain for comparison with previous results [36]”.*

4. Likewise, justify the analysis of TCC instead of Dm28d of the 3rd generation T. cruzi genomes.

**Answer.** The TCC strain has an overall similarity to CL Brener of 99.7% (Berná et al; 2018). In contrast, the Dm28c strain and CL Brener are from different DTUs, thus less similar. For this reason, we choose the 3^rd^ generation sequencing genome data from TCC rather than Dm28c. This reason was already explained in the manuscript, as follow:

Lines 160-163:

*“The first corresponds to the strain used for ChIP experiments, the genome of which was sequenced by second-generation sequencing strategies [18], and the second harbors 99.7% identity to the CL Brener genome and was recently sequenced by PacBio into long reads [21].”*

5. The word “polycistron-CDS” in unusual. May the authors define with accuracy what they mean by it?

**Answer.** Yes, we agree. Thus, we changed all “polycistron-CDS” to “polycistron”.

6. Figure 1. The legend of B and C is confusing. The DNA window of the summary plot is unclear (2kb?, 10kb?, distance start-end?). Please state the size of the region presented in each case.
**Answer.** The DNA window of summary plots is 10 kb. To clarify the legend of Fig 1B and C, we have explained what “Start” and “End” regions mean for each genomic feature.

7. Samples E1 and T1 are similar in the summary plots. Could the authors provide an explanation for this? If the authors have a quantification of the parasite forms in the parasite culture obtained in each sample, it would be worth to include it.

**Answer.** Epi1 has indeed lower H2B.V enrichment compared to E2, and TCT1 has higher H2B.V enrichment than TCT2. However, E1 and TCT1 have different summary plots (compare Epi1 and TCT1 from Fig 1B and C, from S5A and S5B). We believe this difference is due to biological variance among replicates and not due to contaminants. We have a “general rule” in the lab to use only TCT cultures with a maximal of 15% of amastigotes as contaminants. However, for experiments such as Chip-seq and qPCR, we only accept TCTs extracts with a maximal of 5% of amastigotes. We have added a sentence in the Methods section to clarify this part, as follow:

*“For ChIP-seq experiments, TCTs samples with a maximal of 5% of amastigotes were used.”*

8. An analysis of the H2B.V parasites in terms of proliferation and differentiation in comparison to the wild type strain would be useful to evaluate putative alterations of the edited transfectants.

**Answer.** We performed growth curves and evaluated the metacyclogenesis processes in myc-tagged and wild-type parasites (Cas9-transfected parasites). The results are shown in Figure S1D and S1E. H2B.B myc-tagged parasites have similar growth curves to wild-type strains (Figure S1D). The metacyclogenesis was unaffected, as the number of metacyclic trypomastigotes obtained was similar in both conditions (Figure S1D). Together, these data indicated that the genomic edition at H2B.V loci confers no significant phenotypic changes. These data are now cited in the text.

*“Allele-specific PCRs indicate that both alleles were edited (S1B Fig­­). The genome edition neither affects parasite growth nor the metacyclogenesis processes (S1D-E Fig­­). In addition, H2B.V transcript abundance in epimastigotes and TCTs do not differ from wild-type cells or parasites expressing Cas9 (S1F Fig­­). Together, these data indicated that the genomic edition at H2B.V locus confers no significant phenotypic changes.”*

9. Figure S2 B- Amend the color indications.
**Answer.** We corrected the legend of S2B Fig.

10. Line 242-3. Although the authors state “H2B.V interacts with more proteins in TCT”, this is also true for H2B; then it might be the H2B interactome and not the differential role of H2B.V in epimastigotes and TCT the source of variation. Please rephrase to avoid the misleading meaning.

**Answer.** We have rephrased the text as follow:

*“Interestingly, H2B.V and H2B interacts more with proteins found at TCTs than at epimastigote extracts”*

11. Line 277. It is unclear what pull down (our) are the authors referring to in the sentence.

**Answer.** We were referring to the pull-down performed in this paper. We changed the text.

12. Having a myc-H2B.V edited strain, one wonder why the authors did not perform the identification or the validation of protein partners in vivo using the myc tag.

**Answer.** We agree with the referee that we could have performed in vivo IPs using the myc-tagged parasites. However, the myc-tagged parasites (and the ChiP_seq assays) were obtained just recently. Chronologically speaking, we first performed the pull-down assays, then generated the KO parasites and, more recently, obtained the myc-tagged parasites. Nevertheless, we started a similar project to obtain the interactome of H2B.V by IP assays; however, we experienced difficulty in optimizing the IP protocol. Since we have already got the pull-down assays, we preferred to focus our efforts on other experiments, such as the Chip-seq.

13. Figure 3B controls are different for E and TCT. Please clarify the reason.

**Answer.** The controls are the same for life forms; however, it was wrongly assigned in Figure 3B. Two lanes were referred to contain WCE from TCTs, instead of one for each life form. Now, we fixed the figure, and the results of the WB using WCE of epimastigotes and TCTs are shown.

14. The experiment shown in Figure S9 could be in the main article since it is directly related to the hypothesis, and I think would yield statistically significant differences if more replicates are incorporated.

**Answer.** We thank the reviewer for the suggestion; however, we were uncertain about which assay we should incorporate more replicates. Figure S9 has results from 6 different assays. We believe the reviewer was referring to EU or FAIRE assays. We have used three biological replicates for each experiment. In addition, we had performed preliminary experiments to standardize these assays. In neither case, we observed differences between the wt and HtzKO parasites. Since we detected no statistical differences between groups, we would rather keep this Figure on the supplemental part.

Reviewer #2: I will raise some issues which I think should be modified or better addressed in the manuscript:

1. I would suggest modifying the title to: Histone H2B.V demarcates strategic regions in the Trypanosoma cruzi genome and affects parasite differentiation and host cell invasion. Although interesting, the interaction of H2B.V with a bromodomain factor, it was not functionally explored and several other potentially interesting interactions were equally witnessed, so I see no point in including this information in the title.

**Answer.** Both reviewers suggested to change the title. Considering their comments, the title was changed to:

*“****H2B.V demarcates divergent strand-switch regions, some tDNA loci and genome compartments in T. cruzi and affects parasite differentiation and host cell invasion****.”*

2. The authors state that “To improve the data analysis and biological conclusions, the reads were mapped against two T. cruzi genome assemblies (the Esmeraldo-like haplotype of the CL Brener strain and against the TCC 148 strain)”. The comparative analysis was thoroughly described in results, but poorly discussed and a wrap up on the findings and relevance of the comparison is missing in the Discussion.

**Answer.** Yes, we agree. We have wrap up the findings in the Discussion Section, as follow:

*“The comparison of ChIP-seq results in the two genome assemblies obtained by the 2nd (CL Brener strain) or 3rd (TCC strain) generation sequencing strategies reinforces the preferential location of H2B.V at dSSRs, tDNAs, and at the border of the disrupted and conserved compartment. However, this comparison also highlighted differences: more dSSRs harbor no H2B.V enrichment in TCC assembly (68% versus 38% in CL Brener). Among the non-SSR peaks, only 5% of them lay on tDNAs in TCC (compared to 25% in CL Brener). The difference could be related to a better mapping and assembly resolution obtained by the 3rd generation sequencing or by the biological differences between strains. In addition, we detected enrichment of H2B.V in mucins and MASP genes. However, analysis at new assemblies’ genomes indicates that these genes are located preferentially at the first CDS of a given PTU, thus close to dSSRs. These findings strengthen the necessity of comparing the results in the two genome assemblies to assess data reliability and reproducibility. This comparison seems more critical for biological conclusions made at the multigenic family loci, which were better assembled in the 3rd generation sequencing strategies.”*

3. The counterintuitive results, quite interesting and intriguing, on the amount of H2B.V protein versus the profile of deposition on the genome in epimastigotes and TCT should be better explored. The authors focus the discussion on the chromatin structure and histone binding features, association of H2B.V with H2A.Z (as observed in T. brucei), or differences on PTMs (starting on line 367). Possible moonlight activities are mentioned later on in the discussion (line 434), and it seems to me that an unknown function played by this histone variant could be a possible explanation for the abovementioned results. Thus, the connections of their findings and possible moonlight functions should be better explored in the discussion. Moreover, it is necessary to refer to at least one of the several articles presenting the known “moonlight proteins” (or functions) and if histones have ever been shown to harbor moonlight functions.

**Answer.** We believe we have better explored this counterintuitive result in the new version of this article. By sequential salt extraction assay, which evaluated the relative binding affinities of chromatin-associated proteins, we detected that H2B.V has a lower binding affinity to TCT’s chromatin than epimastigotes chromatin. Please, check the answer for reviewer 1 for more detail about this. Then, we have changed the manuscript as follows.

Lines 237-245:

*“… This finding agrees with lower H2B.V staining in myc-tagged TCTs nuclei detected by IFA (S1G Fig) but contrasts with previous findings [31].*

*Thus, we hypothesize that H2B.V may weakly interact with the TCT’s chromatin and be released upon experimental procedures like IFA and ChIP. To address this, we performed a chromatin sequential salt extraction assay, which evaluates the relative binding affinities of chromatin-associated proteins [37]. The results indicated that H2B.V (and H3) is released from chromatin in lower salt concentrations in TCTs than epimastigote forms, suggesting that H2B.V has a lower binding affinity to TCTs chromatin (S6B Fig).”*

We have included a general reference for moonlight proteins/functions and another regarding histones moonlight function. This latter reference describes an intriguing finding that histone tetramer also has cupper reductase activity leading to key evolutionary insights about this protein function origin.

Lines 474-489.

*“Moonlight proteins are multi-tasking proteins that perform various functions [66]. Recently, histone H3-H4 tetramer was shown to have cupper reductase activity, expanding histone function beyond chromatin compaction [67].”*

And

*“Intriguingly, histones are identified on the secretome of T. cruzi [74] and could interfere with infection. Here we found that H2B.V has a lower binding affinity to TCT’s chromatin. However, it remains to be investigated whether H2B.V plays other functions than compaction in TCT’s nuclei and whether it has any other role in parasite infection.”*

4. Finally, the authors forgot to mention that post-transcriptional mechanisms are considered to be the center of gene expression regulation in these parasites. It seems proper to refer the corresponding literature; it would highlight the relevance of their own studies.

**Answer.** We thank the reviewer for this comment. The fact that trypanosome gene regulation relies mainly on post-transcriptional had been mentioned in the previous version (lines 463) however, it had not been cited accordingly. We would rather not discuss the impact of our data on that, as published and unpublished data from our lab (Lima et al; 2021) suggest that chromatin may (in some cases) play a role in gene expression.

**Minor points:**

5. Line 100, page 4 – Leishmania is not italicized.

**Answer.** Done.

Results:
6. Line 162, page 6 – by 2SC would the authors mean 2C?

**Answer.** Please, 2SC means S2C.

7. Lines 164-165. Authors should choose between better explaining the cSSR and tDNA information at this point of the manuscript (including figure panel) or not mentioning it here to leave the information for when presenting the tDNA itself.

**Answer.** We agree with reviewer. This information was duplicated. Thus, we have removed this part from lines 164-165.

8. Line 168, page 6 – there is an s to be removed from S2B-D Fig

**Answer.** Ok.

9. Line 197 – in the sentence “. preferentially located at the first CDS”, wouldn’t it be good to add “of a given PTU”.

**Answer.** We changed to *“ are preferentially located at the first CDSs of a given PTU”*

10. Lines 271-276 – The conclusion on this paragraph is a too strong statement considering that they have only evaluated histone H3.
**Answer.** We changed the text including the sentence.

Lines 298-299

*“Nevertheless, BDF2 interaction with other core histones should be evaluated to confirm this specific interaction.”*

11. Lines317-319 – S9E panel is not presented. Apparently, the S9E panel is presented as S9D, and if the mentioned “decreasing trend” the authors mention refers to panel D, any conclusion would need further experiments/analysis as mentioned in the paragraph that follows (starting on line 320). Thus, I would suggest to either include the mentioned analysis or to present this result as inconclusive, avoiding the bias of mentioning a “decreasing trend” that needs further analysis for a firm conclusion.

**Answer.** Two different figures were wrongly considered as panel S9D. The panel S9E refers to the result of the FAIRE assay, which evaluated the amount of open chromatin in two HtzKO and wild-type cells. This experiment was performed in biological triplicates, but we noticed that the amount DNA recovery upon this strategy varied considerably, especially at the clones. The difference between wt and clones were not statistically significant (T-test: WT x Cl2, p-value=0,2247; WT x Cl4, p-value=0,1746. One-way ANOVA, p-value= 0,3840)) although HtzKO parasites have lesser open chromatin than wt. We agree with the reviewer, and we have removed the term "decreasing trend" to avoid misunderstanding. The sentence was changed to:

Lines 340

*“H2B.V-HtzKO parasites and wild-type parasites have similar levels of active chromatin (S9E Fig).”*

12. S5 Figure is not cited anywhere in Results, only en passant in the Discussion. I suggest inserting the information it contains in the Results section.

**Answer.** We have now cited S5 Fig in the results section. We decided to remove the S5C panel as it is just another example of enrichment of H2B.V in dSSRs. This finding was already shown in Fig 1A, S4A, and S5A. In addition, we have made a few adjustments at S5 Fig to highlight H2B.V localization.

13. S8 Fig. – Panel A description and/or representation must be improved. The colors used (blue, gray, pink and orange) in the lower scheme do not help to understand where they came from.
**Answer.** The reviewer is right. We changed the scheme colors.

Discussion:

14. Lines 368-370 – The sentence should be rephrased.

**Answer.** In light of the results of sequential salt extraction, we have rephrased accordingly.

Lines 406-408

*“Although the H2B.V protein abundance is higher on TCTs, this histone has a lower binding affinity to their chromatin, as observed by sequential salt extraction assays”.*

15. Line 416 – before the i. there is a punctuation missing.

**Answer.** Done.

16. Line 417 – fulfill misses one l

**Answer.** Done.

17. Line 417/418 – The reduced level should go with was
**Answer.** Done.

18. References:
21 among others are bugged – with unrecognized characters

**Answer.** Done.

Legend figures –
19. As a general comment for the legends: in the IGV outputs, frequently, if not always, the authors included a rectangle with interrupted lines. Although the meaning may be obvious, the authors must explain in the legend what these boxes are demarcating.
**Answer.** We added an explanation for all figures (1, 2, S3-5) that contains a rectangle.

20. Fig 1- Improving legend is necessary:
Start-End notation probably corresponds to the S and E indicated at the graph on panels B and C. Does it mean in the case of CDS (B), Start and End of a CDS? And would it indicate start and end of an dSSR or cSSR in panel C?
Text is truncated at line 986.
**Answer.** We have added an explanation about the meaning of S and E. In addition, we removed the truncation.

Lines 1065-1069.

*“Polycistron start and end is related, respectively, to the first ATG (from the first CDS) and the last stop codon (from the last CDS). dSSR and cSSR start refer to the first coordinate downstream, respectively, to the start and end of the left polycistron. dSSR and cSSR end refer to the first coordinate upstream, respectively, to the start and end of the right polycistron.”*

21. Fig 2 – There should be added a “respectively” to the sentence on lines 992-994
**Answer.** Done.

22. S2 Fig.

Line 1049 – Brener is misspelled.
It seems that this legend does not correspond to the S2 figure presented.
**Answer.** The legend had information about peaks also obtained with fold 2. We removed this part from the legend.

23. S4 Fig Text included at the bottom of panel B is truncated.

**Answer.** We have fixed it.
